# Sleep Disordered Breathing in Children with Mitochondrial Disease

**DOI:** 10.1155/2014/467576

**Published:** 2014-12-23

**Authors:** Ricardo A. Mosquera, Mary Kay Koenig, Rahmat B. Adejumo, Justyna Chevallier, S. Shahrukh Hashmi, Sarah E. Mitchell, Susan E. Pacheco, Cindy Jon

**Affiliations:** ^1^Department of Pediatrics, University of Texas Medical School, Houston, TX 77030, USA; ^2^Memorial Hermann Memorial City Hospital, Pediatric Sleep Center, Houston, TX 77030, USA

## Abstract

A retrospective chart review study was performed to determine the presence of sleep disordered breathing (SDB) in children with primary mitochondrial disease (MD). The symptoms, sleep-related breathing, and movement abnormalities are described for 18 subjects (ages 1.5 to 18 years, 61% male) with MD who underwent polysomnography in our pediatric sleep center from 2007 to 2012. Of the 18 subjects with MD, the common indications for polysomnography were excessive somnolence or fatigue (61%, *N* = 11), snoring (44%, *N* = 8), and sleep movement complaints (17%, *N* = 3). Polysomnographic measurements showed SDB in 56% (*N* = 10) (obstructive sleep apnea in 60% (*N* = 6), hypoxemia in 40% (*N* = 4), and sleep hypoventilation in 20% (*N* = 2)). There was a significant association between decreased muscle tone and SDB (*P*: 0.043) as well as obese and overweight status with SDB (*P* = 0.036). SDB is common in subjects with MD. Early detection of SDB, utilizing polysomnography, should be considered to assist in identification of MD patients who may benefit from sleep-related interventions.

## 1. Introduction

SDB is characterized by recurrent, partial, or complete cessation in breathing that disrupts normal sleep. SDB represents a spectrum of disorders that vary from simple snoring to complete upper airway closure as seen in obstructive sleep apnea (OSA) syndrome. SDB may also include central sleep apnea (CSA), prolonged hypoxemia, and hypoventilation.

The prevalence of SDB in children varies from 1.2 to 13.9%, depending on the definition used and the method of data collection [1–4]. OSA syndrome has been estimated to be present in 1 to 2% of healthy children [5] and reports of habitual snoring vary from 4 to 34.5% in the general pediatric population [6–9].

Polysomnography identifies and quantifies sleep disturbances and is the standard method to diagnose SDB [10]. Accurate diagnosis is crucial and therapeutic interventions should be promptly initiated, as untreated SDB is associated with significant morbidity, including somatic growth impairment, poor cognitive and behavioral development, and metabolic and cardiovascular derangements [11].

Primary mitochondrial disorders (MD) are a group of inherited multisystem disorders that impair oxidative phosphorylation and create a deficiency of cellular ATP. The symptoms are varied and many organ systems are involved [12]. Approximately 45% of pediatric patients with MD first present with a neuromuscular problem [13, 14]; however, the character and degree of neuromuscular impairment are poorly defined. Fatigue and hypotonia were the most prevalent symptoms reported in children in the study done by Koene et al. [15]. The association between SDB and neuromuscular disease (NMD) has been reported; 27–62% of children with NMD have SDB [16–19]. Current guidelines recommend that sleep specialists evaluate patients with sleep disturbance and NMD with polysomnography to identify and treat SDB [20–23]. SDB in children with primary MD has not been defined. Although the exact incidence of MD remains unknown, the prevalence of MD is higher than many of the more recognized inherited NMDs, such as Duchenne's muscular dystrophy [12]. Recommendations regarding polysomnography do not exist for patients with MD, even though they may have a high potential to develop SDB. In this study, we describe the polysomnographic findings of sleep disturbances, including SDB, in 18 subjects with primary MD.

## 2. Material and Methods

A retrospective chart review of pediatric patients between the ages of 15 months and 18 years with primary mitochondrial disorder (MD) who were treated at the University of Texas Houston Mitochondrial Center was conducted (Table 1). Patients were diagnosed with MD using the modified Walker criteria [24]. Between 2007 and 2012, 18 subjects with MD who had complete diagnostic polysomnograms secondary to sleep complaints were identified. The modified Epworth sleepiness scale assessed sleepiness but was only documented in 3 patients [25].

Each of the 18 subjects underwent a fully attended nocturnal PSG with videographic monitoring for one night at Memorial Hermann Hospital. Prior to performing the PSG, informed consent and basic past medical history from each subject's parent/guardian were obtained. Height and weight were measured and recorded; tonsillar size and oropharyngeal dimensions using the Mallampati classification system were documented in most patients.

Sleep montage surface electrode sites included F3, F4, C3, C4, O1, O2, M1, M2, right EOG, left EOG, chin EMG, anterior tibialis EMG, and EKG. Snoring was recorded using a snore sensor and audible snoring was noted by the sleep technologist. A thermocouple and nasal pressure transducer recorded nasal and oral airflow. Thoracic and abdominal respiratory effort was measured using impedance plethysmography belts. End tidal carbon dioxide (ETCO2) was measured by capnography via nasal sensors. Oxygen saturation (SpO2) was monitored by a pulse oximeter. All signals were recorded using the Rembrandt (Embla Systems, Kanata, ON, Canada) digital acquisition system.

Each PSG was scored by a sleep technician and then interpreted by a board certified sleep physician. Stages of sleep, respiratory events, arousals, and limb movements were all scored according to the pediatric rules in the 2007* American Academy of Sleep Medicine (AASM) Manual for the Scoring of Sleep and Associated Events* [26]. Apneas were defined as cessation of oral and nasal airflow for at least 2 breaths as compared to the patient's baseline breathing pattern. Apneas were further categorized as obstructive, central, or mixed. Obstructive apneas occurred when there was no airflow for at least 2 breaths with corresponding respiratory effort. Central apneas were identified if there was no airflow or corresponding respiratory effort for ≥20 seconds or at least 2 breaths if there was an associated arousal from sleep, awakening, or ≥3% oxygen desaturation. Mixed apneas were detected if there was no airflow without corresponding respiratory effort which resumed before the end of the apnea. Hypopneas were scored if the peak to trough airflow was decreased by ≥50% for 2 or more breath cycles and the event was accompanied by ≥3% desaturation and/or caused an arousal from sleep. Respiratory effort related arousal (RERA) was scored if there was <50% decrease in the amplitude of nasal pressure signal compared to baseline, flattening of the nasal pressure waveform, accompanying snoring, noisy breathing, elevation in ETCO2, transcutaneous CO2, or visual evidence of increased work of breathing that persisted for ≥2 breath cycles. Periodic limb movements (PLM) were scored if four or more appeared consecutively within a 90-second interval.

The following indices were calculated with respect to the total sleep time (TST) in hours.

- Apnea hypopnea index (AHI) = all apneas + all hypopneas.
- Obstructive AHI = obstructive apneas + mixed apneas + obstructive hypopneas.
- Obstructive apnea index (AI) = obstructive and mixed apneas.
- Central apnea index (CAI) = central apneas.
- Respiratory disturbance index (RDI) = all apneas + all hypopneas + RERA.

OSA was defined using the* International Classification of Sleep Disorders 2nd Edition (ICSD-2)* [27]. Central sleep apnea (CSA) was identified when CAI was >3 events per hour of TST [28]; sleep hypoventilation was detected when ETCO2 > 50 torr for >25% of TST [26]; hypoxemia was defined as oxygen saturation <90% for >2% of TST [29]. SDB was present if there was a respiratory disturbance index (RDI) of ≥5 events per hour of TST or presence of OSA, CSA, sleep hypoventilation, or sleep hypoxemia [30]. Periodic limb movement (PLM) index was relevant if there were >5 PLMs per hour of TST [26]. Primary snoring was diagnosed if the patient demonstrated snoring during the PSG in the absence of SDB.

Muscle tone is defined as the muscle's resistance to passive stretch during the resting state. Tone helps to maintain posture and multiple disease states can alter muscle tone. Low tone is a decreased resistance to passive motion and high tone is an increased resistance to passive motion. Tone was assessed in our study population by a board certified pediatric neurologist. The observed tone of each subject was classified into three groups: hypotonia (low tone), normal tone, and hypertonia (increased tone). Results are presented in Table 1.

Ten patients had spirometry testing (Breeze Suite 6.4, Medical Graphics Corporation, St. Paul, MN), as per American Thoracic Society guidelines [31], within a 12-month period from the polysomnography. Predicted normative values were based on Knudson et al. published data [32]. Spirometry results were interpreted by two pediatric pulmonologists, as having presence of a normal, obstructive, restrictive, or mixed restrictive/obstructive airflow pattern.

The MD patients analyzed in this study were referred to The University of Texas Medical School Houston, Division of Pulmonary Medicine by the University's Mitochondrial Center. This study was approved at inception by the Committee for the Protection of Human Subjects (CPHS# HSC-MS-13-0622).

### 2.1. Analysis

Frequencies with percentages were calculated for all categorical variables. Continuous variables were described using means (with standard deviation, SD) or medians (with interquartile ranges, IQR) for normal and nonnormal data distributions, respectively. Categorical variables were compared using Fisher exact test or chi-square test, as appropriate. All statistics were performed using STATA v.12 (College Station, TX). Statistical significance was assumed at a type I error rate of 0.05.

## 3. Results

### 3.1. Symptoms

The common symptoms prompting polysomnography were parental or self-reported excessive daytime somnolence/fatigue in 11 (61%), snoring in 8 (44%), and sleep movement in 3 (17%) subjects (Table 2). Sleep movement complaints consisted of muscle jerks. Of the 8 subjects that reported snoring, only 4 were corroborated by polysomnography.

### 3.2. Sleep Architecture

The average total sleep time (TST) and total test time (TTT) were 6.8 hours (SD: 0.7 hours) and 8.1 hours (0.6 hours), respectively. The median sleep stage time as percentage of TST was 3.1% (IQR: 1.8–4.6) for stage N1 sleep, 53.5% (IQR: 44.1–61.8) for stage N2 sleep, 22.8% (IQR: 20.0–30.3) for stage N3 sleep, and 16.6% (1.8–22.3) for rapid eye movement (REM) sleep.

### 3.3. Respiratory Events

**Figure 1:**
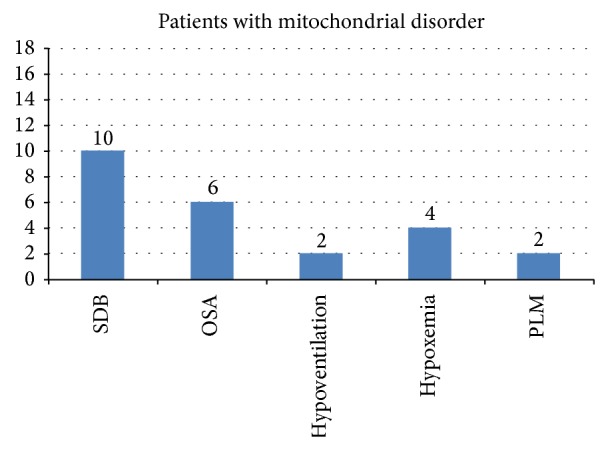
Sleep disordered breathing characteristics in 18 patients with mitochondrial disorders.

In total, 10 (56%) subjects had SDB due to either presence of OSA, CSA, hypoventilation, and hypoxemia or an RDI ≥ 5 (Figure 1). The 2 subjects with the highest RDI (47.9/hour and 10.4/hour) had a complex I mitochondrial defect. The only patient with Leigh syndrome had sleep hypoxemia.

Of the 10 subjects with MD and SDB, 6 demonstrated (60%) OSA (Figure 1) with an average obstructive AHI of 2.7/hour with an obstructive AI of 0.7/hour. There were no patients with CSA, since all patients had CAI that was less than 1.4 per hour. Four subjects with SDB (40%) were noted to have sleep hypoxemia with an average of 5.1% of TST < 90% saturation.

Based on 2007 AASM scoring criteria [26], 2 subjects with SDB (20%) demonstrated sleep hypoventilation with ETCO2 > 50 torr for >25% of TST. There were 3 other children with elevated ETCO2 (>50 torr) that was not diagnostic for sleep hypoventilation but was thought to contribute to their symptoms. Subject 13 had elevated ETCO2 for 22.8% of TST, subject 2 had elevated ETCO2 for 17.6% of TST, and subject 14's ETCO2 was elevated for 10.5% of TST.

Of the 5 patients with clinical history of allergic rhinitis, none were diagnosed with SDB. There were 5 subjects with enlarged tonsils, of which only 2 were considered to have SDB. Of the 3 subjects who had previously had an adenotonsillectomy, 2 had SDB. Of note, both of the subjects who continued with SDB after their adenotonsillectomy were obese.

Based on the Centers for Disease Control (CDC) definition of obesity (BMI of >95 percentile for age) and overweight (BMI of 85–95 percentile for age) in children, 4 (22%) of the patients were designated as obese and 1 (6%) was overweight. All of the obese and overweight patients were diagnosed with SDB. Two (11%) were noted to be underweight with BMI of <3% for age, of which 1 had SDB.

### 3.4. Movement Events

Two subjects (11%) were found to have elevated PLMI > 5/hour. Neither had sleep movement complaints as indicators for polysomnography.

### 3.5. Muscle Tone

Twelve of the 18 subjects (67%) who underwent PSG had abnormal muscle tone (Table 1). Of the 10 patients with SDB, 9 (90%) had abnormal tone. Specifically, 6 had hypotonia and 3 had hypertonia. There was a significant association between abnormal muscle tone and SDB in the study (*P* value = 0.043).

### 3.6. Spirometry

Ten patients had available spirometric data, of which 8 were noted to have normal airflow pattern, 1 was noted to have restrictive pattern, and 1 was noted to have mixed restrictive/obstructive airflow pattern. Of the 10 patients who underwent spirometry, 4 patients had SDB, all of which were noted to have normal airflow patterns.

## 4. Discussion

As expected, in the current study, we found a high incidence of SDB in children with MD undergoing nocturnal PSG. SDB was present in 56% of the subjects, compared to 1.2–13.9% in general population [1]. The most common SDB finding was OSA (60%), followed by hypoxemia (40%) and sleep hypoventilation (20%). In addition, elevated PLMI was detected in 11% of the subjects. The basis of this finding could be the result of reduced activity of respiratory muscles during sleep or an intrinsic disorder that has not been characterized in the study population. To the best of our knowledge, this is the first study that describes SDB in children with MD.

OSA was found in 60% of subjects with SDB and MD. In this study, pediatric OSA was designated using the ICSD-2 definition [27]. The authors contend that an obstructive AHI of ≥1 per hour may overestimate the incidence of OSA, especially since the threshold of clinically significant OSA in children has not been established. Recently, the Childhood Adenotonsillectomy Trial (CHAT) defined the threshold for OSA as an obstructive AI of ≥1 event per hour or obstructive AHI of ≥2 events per hour [33]. CHAT is the first large randomized controlled trial in the children designed to address the effectiveness of adenotonsillectomy in children with OSA syndrome in regard to polysomnographic, neurocognitive, and behavioral outcomes. If we applied the CHAT OSA definition to our patient population, OSA was detected in only 3 (30%) of the subjects with SDB and MD and the mean obstructive AHI changed from 2.7 per hour to 4.1 per hour. With either definition, the obstructive AHI was <5 per hour which is considered mild [10].

Sleep hypoventilation, defined according to the 2007 AASM criteria with ETCO2 > 50 torr for >25% of total sleep time, was found in two subjects (22%). Elevated ETCO2 (>50 torr), which did not meet the threshold for the AASM definition for sleep hypoventilation, was found in 3 subjects. Marcus et al. [34] demonstrated that children were more prone to hypoventilate than adults since a child's upper airway was more resistant to full closure with increasing applied negative pressure. Other factors may lead to hypoventilation, such as obesity, adenotonsillar enlargement, and abnormal muscle tone. Of the 5 patients with elevated ETCO2, 2 were obese, none had tonsillar hypertrophy, and 4 had hypotonia. As such, abnormal muscle tone may be an important component of sleep hypoventilation in children with MD and SDB. Of note, 3 of the patients with elevated ETCO2 had spirometric measurements which were normal. Interestingly, there were no subjects with CSA, despite a relatively low CSA threshold (CAI > 3).

To date, sleep hypoxemia has not been clearly defined in the pediatric population. The Centers for Medicare and Medicaid Services, a national social insurance program for Americans who are older than 65 years of age or are disabled, defines hypoxemia based on polysomnography in adults as SpO2 < 88% for more than 10 minutes. For this study, we used the CHAT definition, which established the diagnostic threshold at SpO2 < 90% for 2% of total sleep time [33]. Four subjects (40%) had sleep hypoxemia, one of which was placed on supplemental oxygen during the sleep study.

Oxygen is indispensable for the survival of all organisms due to its role in the production of ATP by mitochondria. Functional and quantitative mitochondrial dysfunction inherent to the MD could be potentially further deteriorated by SDB. Reactive oxygen species (ROS) are chemically reactive molecules containing oxygen formed as a natural byproduct of cellular metabolism. At physiologic levels, ROS play an important role in cell signaling and homeostasis but unregulated ROS can damage cell structures and incite apoptosis. Our cells have antioxidants to neutralize reactive oxygen species (ROS) produced during normal cellular functions. However, stressors such as infection, hypoxemia, and SDB increase ROS production. High levels of ROS can overwhelm the innate antioxidant capacity leading to mitochondrial dysfunction. During episodes of hypoxia, ROS function in signaling O2 supply increments to decrease oxygen consumption via AMPK activation and endocytosis. Hypoxia-inducible factors orchestrate transcriptional responses also by promoting erythropoietin expression, augmentation of RBC production, and maintenance of ATP levels. Moreover, stimulation of vascular endothelial growth factor prompted by hypoxia leads to angiogenesis in undersupplied tissues. Therefore, shifts in quantity of ROS in either direction could alter the fragile homeostatic balance leading to pathologic alterations and disease [35]. OSA in itself may increase ROS, therefore worsening MD.

In this study, SDB was defined by the presence of OSA, CSA, hypoxemia, hypoventilation, and/or RDI > 5 per hour. In the literature, the prevalence of SDB in children varies from 1.2 to 13.9%, due to inconsistent definitions and the method of data collection [1–4]. The authors agree there is a wide and discrepant range of definitions for SDB which made a specific or concise cutoff difficult. Most publications agree that SDB encompasses OSA, CSA, hypoxemia, and hypoventilation. The authors chose RDI of >5/hour to capture presence of sleep disordered breathing, aside from the more well-defined OSA, CSA, hypoventilation, and hypoxemia. Tang et al. [30] showed that, using an RDI threshold of RDI ≥ 5/hour, the prevalence of SDB varied less (i.e., by <10%) when compared to using RDI ≤ 3/hour (varied by 1–74%).

There are multiple reasons for children with MD to develop SDB: neuromuscular abnormalities, tonsillar hypertrophy, obesity, allergic rhinitis, or the use of sedative medications [17, 36, 37]. We found inconsistent correlation between adenotonsillar hypertrophy, posttonsillectomy status, allergic rhinitis history, and SDB. Abnormal growth parameter was associated with SDB, especially when the BMI was >85% for age. OSA syndrome is estimated to be present in approximately 13–59% of obese children in the general population [38]. Obesity may contribute to upper airway narrowing due to fat deposition and restrictive respiratory function due to truncal adipose tissue deposition. In our dataset, there was an association between obesity and SDB (*P* = 0.036), with all 5 (27%, overall) of the obese patients having SDB. There was no association between obesity and abnormal muscle tone (*P* = 0.114) and the small sample size precluded any multivariate analysis. In addition, 2 out of the 3 SDB patients who had a positive history of adenotonsillectomy were obese. This is consistent with current findings in the general pediatric obese population that OSA may persist despite adenotonsillectomy [39, 40]. Excessive weight gain may be a contributing factor to development of SDB in our patient population. Two of the subjects were underweight with inconsistent association with SDB. Previous studies have also supported the fact that, contrary to the dogma that mitochondrial patients are typically malnourished, about one-third is obese [41].

Sedative medications were excluded as a contributing factor to the SDB in our study population as none of our study subjects were on such medications during the period preceding their sleep studies. Of note, fourteen out of our eighteen subjects that had an echocardiogram done within a year of their sleep study had no features suggestive of a cardiomyopathy.

The prevalence of SDB in MD has not been established. Untreated, SDB may lead to significant morbidity and increased mortality. The current study found a significant association between SDB and abnormal muscle tone in our subjects with MD. Predisposing factors to SDB in NMD include reduced ventilatory responses, reduced activity of respiratory muscles during sleep, and poor lung mechanics due to their underlying neuromuscular disorder. However, some patients with mitochondrial disease do not have neuromuscular disease or it is subtle.

Normally, during REM sleep atonia, the activity of the intercostal muscles is reduced but diaphragmatic muscle function remains intact. This reduction is compensated during awakening and non-REM sleep by the intercostal and accessory muscles of ventilation [42]. In neuromuscular diseases, there is a disruption of diaphragmatic ventilation [36]. Furthermore, subjects with NMD may present with a restrictive lung disorder as a consequence of chest muscle weakness, as well as obstructive respiratory function alterations, due to the weakness or collapse of the pharyngeal muscles. On the other hand, unlike the current study, in generalized myopathies, such as Duchenne's muscular dystrophy, sleep-related ventilatory deficiency is found exclusively in terminal stages of the disease [43]. Further cohort studies are necessary to establish if SDB is more prevalent in patients with MD regardless of the severity of neuromuscular weakness inherent to the pathophysiology of MD.

Ten patients had spirometry data available. Due to the retrospective nature of the study, the timing of spirometric testing was variably relative to the date of the PSG. A few of the spirometry tests were done within days of the PSG and others were within 1 year of the PSG. As such, the available spirometric measurements may not reflect the patient's true respiratory function at the time of the PSG. In addition, the younger subjects were not able to perform the technically difficult spirometric maneuvers, which limited the available data. Regardless, the 4 patients with spirometry measurements and SDB had normal airflow patterns which suggest that the SDB may predominantly be a sleep phenomenon and does not seem to translate to daytime respiratory impairment.

There is a significant overlap of symptoms between sleep disordered breathing (SDB) and mitochondrial disease (MD). Nonspecific complaints such as fatigue and lethargy, weakness, and learning disabilities found in both disorders make it almost impossible to distinguish SDB from MD without a polysomnography. The increased incidence of excessive daytime somnolence seen in study patients (61%) compared to the general population (20%) [19] suggests that although contributory, the hypersomnolence is more likely a manifestation of the MD than a pure reflection of the SDB. Modified Epworth sleepiness scale scores were found only in 3 of the patients who underwent PSG but did not have SDB. As such, objective measures of excessive daytime somnolence were not available. We can however speculate that symptoms associated with SDB aggravate the overall decreased daytime stamina seen in many patients with MD. Due to the retrospective nature of the study, the level of parent or patient reported symptoms could not be clarified or quantified. This association between SDB and mitochondrial disorder remains unclear.

In summary, we found that SDB constitutes a frequent finding in children with MD, compared to the general population. Clinical history is not a helpful screening tool in identifying SDB due to nonspecific complaints and overlap of symptoms between these two diseases. Polysomnography should be considered in all children with MD to avoid further potential deterioration caused by SDB comorbidity. Children with MD who have abnormal tone and/or are obese/overweight may be more prone to SDB and may need more careful screening for SDB. Moreover, MD may be added in spectrum of differential diagnosis in all children with nonspecific multiorgan complaints and SDB. The obvious limitation of this study is a small sample size and retrospective design of the study. Larger cohort studies are needed to further explore the association between SDB and MD in the pediatric population.

## Figures and Tables

**Table 1:** Patient demographics, allergic rhinitis history, adenotonsillar history, and neuromuscular tone characteristics in patients with mitochondrial disorder.

ID	Gender	Age (years) at PSG	Race/ethnicity	BMI in kg/m^2^ (percentile)	Allergic rhinitis by history	Tonsil & adenoid history	NM Tone	Spirometry
1	F	9	W NH	17.2 (50–75%)	Y	2+	Normal	Mixed
2	M	6	W NH	15.5 (75%)	N	N	Normal	Normal
3	M	3	W NH	13.3 (90%)	N	4+, A	Hypotonia	Normal
5	M	2	W NH	12.3 (<3%)	Y	3+	Normal	—
6	F	12	W NH	26.8 (95–97%)	Y	T & A	Hypotonia	Normal
7	M	16	W NH	23.7 (75–85%)	Y	N	Hypotonia	Normal
8	M	10	W NH	18.7 (75%)	N	T & A	Normal	—
9	M	2	W NH	19.4 (>97%)	N	N	Hypertonia	—
10	F	2	W NH	15.4 (25%)	N	Y	Hypotonia	—
11	M	5	W NH	14.2 (10%)	N	Y	Normal	Normal
12	M	7	W NH	29.6 (>97%)	N	N	Hypotonia	Normal
13	F	5	W Hispanic	22.0 (>97%)	N	T & A	Hypotonia	Normal
14	M	1.5	W NH	18.8	N	N	Severe hypotonia	—
15	F	3.5	W NH	15.7 (50%)	N	N	Hypertonia	—
16	F	2	W Hispanic	16.3 (50%)	N	N	Hypertonia with cogwheel	—
17	M	16	W NH	15.8 (<3%)	N	N	Normal	—
18	M	18	W NH	22.5 (50%)	N	N	Hypotonia	Restrictive
19	F	11	W NH	18.0 (50%)	Y	N	Hypotonia	Normal

PSG: polysomnogram, W: white, NH: non-Hispanic, H: Hispanic, BMI: body mass index, T & A: posttonsillectomy, A: postadenoidectomy, NM: neuromuscular, MD: mitochondrial disorder, Y: yes, N: no or none, and ESS: Epworth sleepiness scale.

**Table 2:** Polysomnographic indications, test time, respiratory data, and limb movement description in patients with mitochondrial disorder.

ID	PSG indication	TTT	TST	Snoring	Obstructive AI	Obstructive AHI	CAI	Total AHI	RDI	% TST ETCO2 >50 torr	% TST SpO2 <90%	PLM index
1	Snoring, EDS	8.0	7.0	N	0	0	0.3	0.3	0.3	0	0	0
2	EDS, inattention at school, snoring	8.2	6.7	Y	0	0.9	0.5	1.4	1.6	17.6	0.8	5.6
3	EDS, inattention at school	9.1	5.1	N	0.2	0.2	0	0.2	6.3	0	0	0
5	Restless sleep	8.0	7.2	N	0	0	0.4	0.4	0.4	0	0.1	0
6	EDS	7.9	6.3	N	0.3	0.6	0	0.6	1.9	97.2	0.1	0
7	EDS	7.7	7.2	N	0	0.4	0.3	0.7	0.7	0	0.2	0
8	Snoring, witnessed apneas, inattention at school	8.1	6.8	N	0	0	0.3	0.3	0.3	0.4	0.1	0
9	Frequent nighttime awakening	8.2	6.9	Y	0.1	1.3	0.1	1.5	1.6	0.1	0.2	0
10	Frequent night wakening and muscle jerking episodes	7.6	6.4	N	0	0.5	1.3	1.7	2.5	0	2.7	0
11	EDS	7.4	6.6	Y	0	0.2	0.5	0.6	0.6	0	0.2	0
12	EDS, inattention at school, snoring	8.9	7.6	N	0	3.0	0.1	3.2	4.6	0.1	0	0
13	Snoring, gasping during sleep	8.9	7.9	Y	0.3	4.5	0	4.7	5.0	22.8	7.5	22.6
14	Restless sleep, muscle jerks, heavy breathing	8.6	8.1	N	0	0.6	0.7	1.4	1.6	10.5	2.9	0
15	Difficulty sleeping, EDS	7.6	7.2	Y	2.8	4.7	0.7	5.4	10.4	25.2	0.1	0
16	Snoring	7.3	5.7	N	0.9	1.1	0.2	1.2	47.9	0.4	7.4	0
17	EDS, fatigue	7.2	6.4	N	0.3	1.4	0.3	1.7	13.6	0	0.6	0
18	Snoring, morning headaches, EDS	7.2	6.4	Y	0	0.3	0.2	0.3	1.4	0	0	0
19	Snoring, morning headaches, EDS	8.5	7.8	Y	0.1	0.1	0	0.1	0.4	0	0	0.8

PSG: polysomnogram, TTT: total test time, TST: total sleep time, AI: apnea index, AHI: apnea hypopnea index, CAI: central apnea index, RDI: respiratory disturbance index, PLM: periodic limb movement, EDS: excessive daytime somnolence, Y: yes, and N: no.
